# Situating Machine Intelligence Within the Cognitive Ecology of the Internet

**DOI:** 10.1007/s11023-016-9416-z

**Published:** 2017-01-03

**Authors:** Paul Smart

**Affiliations:** Electronics and Computer Science, University of Southampton, Highfield, Southampton SO17 1BJ UK

**Keywords:** Machine intelligence, Machine learning, Artificial intelligence, Internet, Situated cognition, World Wide Web

## Abstract

The Internet is an important focus of attention for the philosophy of mind and cognitive science communities. This is partly because the Internet serves as an important part of the material environment in which a broad array of human cognitive and epistemic activities are situated. The Internet can thus be seen as an important part of the ‘cognitive ecology’ that helps to shape, support and (on occasion) realize aspects of human cognizing. Much of the previous philosophical work in this area has sought to analyze the cognitive significance of the Internet from the perspective of human cognition. There has, as such, been little effort to assess the cognitive significance of the Internet from the perspective of ‘machine cognition’. This is unfortunate, because the Internet is likely to exert a significant influence on the shape of machine intelligence. The present paper attempts to evaluate the extent to which the Internet serves as a form of cognitive ecology for synthetic (machine-based) forms of intelligence. In particular, the phenomenon of Internet-situated machine intelligence is analyzed from the perspective of a number of approaches that are typically subsumed under the heading of situated cognition. These include extended, embedded, scaffolded and embodied approaches to cognition. For each of these approaches, the Internet is shown to be of potential relevance to the development and operation of machine-based cognitive capabilities. Such insights help us to appreciate the role of the Internet in advancing the current state-of-the-art in machine intelligence.

## Introduction

Over the past two decades the Internet^1^ has emerged as an important part of the material environment in which an ever-expanding array of human cognitive and epistemic activities are situated. This makes the Internet a system of considerable importance to the philosophy of mind and cognitive science communities. Fueled by developments in wireless technology and the emergence of a plethora of Internet-enabled devices (e.g., smartphones), the Internet has arguably become a critical element of our cognitively-relevant environment or ‘cognitive ecology’. Many kinds of cognitive tasks, not to mention an increasing number of social activities, rely on the Internet at one point or another, and this makes the Internet a critical focus of attention for those who see environmental (i.e., extra-neural and extra-organismic) factors as playing an explanatorily-significant role in human cognizing (e.g., Clark 2008).

The idea that we should view the Internet as a form of cognitive ecology is inspired by so-called ecological approaches to cognition (Bateson 1972; Malafouris 2013; Hutchins 2010; Tribble and Sutton 2011; Neisser 1997; Cooke et al. 2004; Hirose 2002; Barrett 2011). These approaches emphasize the role of the extra-neural environment in shaping, supporting, and perhaps even realizing human cognitive states and processes. Hutchins (2010), for example, embraces an ecological approach to cognition when he suggests that our attempt to understand “cognitive phenomena must include a consideration of the environments in which cognitive processes develop and operate” (p. 706). In the absence of this sort of wider ‘ecological’ analysis, Hutchins fears that we may lose sight of the forces and factors that undergird our (human) cognitive capabilities. In this respect, Hutchins expresses a view that is broadly consistent with approaches that are typically subsumed under the heading of situated cognition (Robbins and Aydede 2009a).

A number of bodies of existing work (spread across a range of disciplines) appeal to the idea that the Internet serves as a form of cognitive ecology for human cognition. The idea has perhaps its most explicit expression in a paper by Smart et al. (forthcoming). In this case, the appeal to a cognitive ecological conceptualization of the Internet is used to motivate a situated approach to the analysis of human-Internet interactions. In particular, Smart et al. (forthcoming) seek to assess the cognitive significance of the Internet by viewing human-Internet interactions from the perspective of extended (Clark 2008; Clark and Chalmers 1998), embedded (Rupert 2004, 2009), embodied (Shapiro 2007, 2011, 2014; Anderson 2003), and collective/distributed (Theiner 2014; Theiner et al. 2010; Hutchins 2001, 1995) approaches to cognition. Similar sorts of analyses are provided by a growing body of work in philosophy and cognitive science (Staley 2014; Halpin 2013; Halpin et al. 2010; Smart 2012, 2013, 2014, forthcoming; Clowes 2015).

Despite the fact that the Internet is an increasingly popular target of philosophical analysis, there is one respect in which the existing literature is somewhat lacking. This shortcoming relates to the almost exclusive focus on human cognition. Such a focus is, of course, understandable given the nature of our interaction with (and increasing dependence upon) the contemporary Internet. The focus is, however, unfortunate, because there is considerable evidence to suggest that the Internet is poised to exert a significant influence on machine-based cognitive capabilities.^2^ We can get a feel for the nature of this influence by reflecting on the way in which the Internet has influenced research into machine learning and Artificial Intelligence (AI). We thus see increasing amounts of research devoted to cognitive computing systems (Hurwitz et al. 2015; Kelly and Hamm 2013), deep learning algorithms (Najafabadi et al. 2015), big data analytics (Hurwitz et al. 2015; O’Leary 2013), the Semantic Web (Berners-Lee et al. 2001), and so on. The Internet has also spawned a renewed interest in the familiar idea of human–machine symbiosis (e.g., Licklider 1960; Jacucci et al. 2014) where human and machine capabilities are factored into hybrid processing loops that span the biological and technological domains. Such interest is evidenced by the recent explosion of research into ‘human-in-the-loop’ systems (Branson et al. 2014), game-powered machine learning systems (Barrington et al. 2012), human computation systems (Law and von Ahn 2011; Michelucci 2013), citizen science platforms (Khatib et al. 2011a) and Internet-enabled forms of collective intelligence (Michelucci and Dickinson 2016). This efflorescence of research relating to Internet-enabled forms of machine intelligence is largely overlooked by existing attempts to assess the cognitive significance of the Internet. And yet, when it comes to cognitive capabilities, it seems that the Internet is likely to be just as relevant to machine cognition as it is to human cognition (perhaps even more so).

In view of all this, I suggest that we should see the Internet as an environment that is relevant to the cognitive capabilities of *both* human and machine agents. Such a view encourages us to assess the cognitive impacts of the Internet from the conceptual standpoint of situated cognition (Robbins and Aydede 2009b).^3^ In what follows, therefore, I will seek to explore the way in which the notions of extended, embedded, scaffolded, and embodied cognition can be used to help us gain a better understanding of online or Internet-situated forms of machine intelligence.^4^ Not only does this approach help to establish an important and interesting parallel with work that focuses almost exclusively on human agents, it also helps, I suggest, to reveal a number of important directions for research that spans the disciplines of computer, cognitive and social science (see Sect. 7).

The structure of the present paper is organized around the topics of extended, embedded, scaffolded and embodied cognition. Section 2 introduces the notion of extended cognition (Clark and Chalmers 1998; Clark 2008; Menary 2010) and applies it to the specific case of Internet-situated forms of machine intelligence. The result is a claim about the potential role of the Internet in supporting a novel form of cognitive extension. In particular, it is suggested that the Internet enables the human social environment to be incorporated into the processing loops that realize machine-based cognitive capabilities. Section 3 assesses the cognitive impacts of the Internet from the perspective of embedded cognition (Rupert 2004, 2009). In this case, human agents are seen to play an important role in shaping and supporting machine-based cognitive capabilities, without thereby serving as constituent elements of the physical machinery that realizes those capabilities. Section 4 extends the analysis to the case of scaffolded cognition (Sterelny 2010). Scaffolded cognition is presented as the idea that extra-agential resources (i.e., physical elements that exist as part of an agent’s environment) can play an important role in the *development* of cognitive capabilities. When applied to the case of machine cognition, it is claimed that the Internet enables human agents to play an important role in scaffolding the development of machine-based cognitive capabilities. Section 5 attempts to assess the cognitive significance and relevance of the Internet from the perspective of embodied cognition (Shapiro 2011, 2014). In this case, it is suggested that we can see the Internet as enabling the human social environment, as well as ever-increasing array of Internet-enabled devices, to function as literal body parts. Finally, Sect. 6 discusses the extent to which human-Internet interactions can be seen as a form of ‘ecological engineering’ (Sterelny 2003) or ‘cognitive niche construction’ (Clark 2008). The basic idea, in this case, is that we can see the Internet as a means of enabling humanity to play a productive role in the creation and configuration of a cognitively-potent online environment, one that is poised to shape the profile of both human and machine cognition.

## Extended Cognition

The term ‘extended cognition’ identifies an important and influential body of work within the philosophy of mind (see Clark 2008). At its core is the claim that the causally-active physical vehicles of cognition can, at least in principle, extend beyond the biological borders of the individual to include a range of extra-organismic resources (Clark 2008; Clark and Chalmers 1998).

Although the notion of extended cognition is intended to challenge our bio- and neuro-centric intuitions (and prejudices) regarding the material bases of intelligence, there is a sense in which the existing philosophical debate remains skewed towards the biological realm. We see this in the way in which practically all discussions of extended cognition limit their attention to the case of human (or at least biological) intelligence. In the majority of cases, therefore, what emerges as the central focus of philosophical debate is the issue of whether *human* cognition is materially extended as a result of some form of bio-artefactual or bio-technological bonding. This focus is, of course, understandable, given the inherent interest in explaining the rather distinctive forms of cognitive success that are the hallmark of our species. It is, however, a focus that is challenged by the increasing sophistication of machine learning systems and cognitive computing platforms. And it is further challenged, I would argue, once we situate machine intelligence within the cognitive ecology of the Internet.

The idea that I want to canvass here is called Human-Extended Machine Cognition (HEMC). As its name suggests, HEMC is a form of extended cognition. However, rather than being centered on an individual human agent, HEMC is a form of extended cognition that is centered on a synthetic intelligent system. HEMC is, in essence, the idea of materially-extended machine cognition. In particular, it is a form of extended cognition in which the relevant information processing loops extend beyond the realms of machine-based computational processing to include the contributions of one or more human agents. Whereas conventional cases of extended cognition oblige us to focus on the individual human agent and consider how their cognitive states and processes might be extended by virtue of their interactions with a surrounding nimbus of non-biological elements, the HEMC concept encourages us to engage in something of a ‘mental flip’, wherein we focus our attention on the way in which the cognitive capabilities of a machine-based system (e.g., a cognitive computing platform) are realized, at least in part, by a more-or-less permanent penumbra of human individuals.

Inasmuch as we accept the basic possibility of HEMC, we should obviously consider the extent to which such forms of cognitive extension are materially possible. Although it should be clear that nothing prevents important and compelling cases of HEMC arising outside the socio-technical context of the Internet,^5^ the Internet affords an environment that is particularly apt to support the practical realization of HEMC-based systems. One reason for this concerns the growth and popularity of the Internet. Crucially, the Internet has emerged as a major part of the technological fabric associated with contemporary society. It is, as such, a system that is used by an increasing proportion of the World’s population. The result is that we can see the Internet as providing access to significant numbers of cognitively-sophisticated processing units (i.e., individual human agents). Although we might question the extent to which the elements of this large-scale social environment are suitably poised to participate in episodes of machine-based processing,^6^ what should not be in doubt is the fact that the Internet provides the basis for large-scale forms of social participation in a variety of online tasks (see Kraut et al. 2010). Indeed, existing work in the area of human computation (Law and von Ahn 2011; Michelucci 2013) already attests to the possibility of ‘incorporating’ human individuals into bio-technologically hybrid processing loops.

There are a number of reasons why the HEMC concept is worthy of further philosophical and cognitive scientific scrutiny. Firstly, the concept serves to bridge two currently disparate areas of intellectual inquiry. One of these areas is, of course, delineated by existing philosophical debates concerning the notions of extended cognition and the extended mind. The other area emerges in relation to research on Internet-enabled forms of human–machine cooperation (Smart and Shadbolt 2014; Michelucci 2016). This includes, for example, a considerable body of work that has emerged in relation to human computation systems (Law and von Ahn 2011; Michelucci 2013) and citizen science platforms (Lintott and Reed 2013). Indeed, when reading the literature on human computation, one sometimes encounters an almost implicit reference to the idea of multiple (human) individuals serving as part of the realization base for the processing capabilities a diverse array of emerging socio-computational systems (see Michelucci 2016; Michelucci and Dickinson 2016). The HEMC concept provides a means of making this idea explicit while simultaneously establishing an important point of conceptual contact with recent work in the philosophy of mind (Clark 2008).

Another reason why the HEMC concept is important is because of its potential to illuminate novel approaches to the implementation of intelligent systems. Perhaps one of the most important considerations here relates to issues of complementarity. It has long been recognized that one of the virtues of cognitive extension is its ability to harness the complementary contributions of both biological and non-biological resources (Sutton 2010; Wilson and Clark 2009). Wilson and Clark (2009), for example, note the importance of extended cognition in yielding “hybrid processes in which the inner and the outer contributions are typically highly distinct in nature, yet deeply integrated and complementary” (p. 72). An appreciation of such complementarity is, of course, also evident in much of the literature pertaining to machine intelligence, especially work that goes under the heading of complementary computing (Kapoor et al. 2008), mixed-initiative systems (Horvitz 2007), interactive machine learning (Fogarty et al. 2008) and heterotic computing (Kendon et al. 2015). There is, however, a sense in which the notion of HEMC adds something new to this debate on complementarity. This is the idea—often encountered in the extended mind literature—that the exploitation of complementary, bio-external resources may be of crucial relevance in accounting for some of the more advanced aspects of human cognition. As Clark (2003) notes, “what is special about human brains, and what best explains the distinctive features of human intelligence, is precisely their ability to enter into deep and complex relationships with non-biological, props, and aids” (p. 5). Such claims should encourage the designers of intelligent systems to give serious thought to issues of hybridization and complementarity. For inasmuch as human cognition is grounded in the ability to assemble and exploit processing loops that extend across the brain, the body and the world, then perhaps it is somewhat unfair to expect a system that trades in only one kind of information processing economy (e.g., a connectionist or classical rule-and-symbol processing regime) to achieve the kinds of cognitive success that we deem to be representative of human-level intelligence. Put another way, if hybridization is a fundamental feature of the human cognitive architecture, then perhaps our best approach to engineering intelligent systems is to rely on solutions that factor in the representational and computational contributions of elements that are drawn from *both* the biological and non-biological domains. Just as advanced forms of human intelligence are mostly (and possibly always) grounded in networks of bio-artefactual and bio-technological coupling, so too perhaps the route to advanced forms of machine intelligence lies in an ability to merge the modes of representation and processing made available by both our biological brains and our current arsenal of non-biological computing systems.

## Embedded Cognition

According to those who embrace the notion of extended cognition, human cognitive processes sometimes supervene on elements that lie outwith the biological borders of the individual. This is clearly a somewhat radical-sounding proposal. A more conservative view comes in the form of embedded cognition, which is championed by the likes of Rupert (2004, 2009). In contrast to the idea that extra-organismic elements play a constitutive role in the realization of cognitive states and processes, Rupert suggests that we should see extra-organismic (or extra-agential) resources as merely influencing inner, biologically-based(?) cognitive routines. This is not to say that advocates of embedded cognition do not recognize the importance of extra-organismic resources in shaping the course of cognitive processing. As Rupert (2004) himself notes:

...cognitive processes depend very heavily, in hitherto unexpected ways, on organismically external props and devices and on the structure of the external environment in which cognition takes place. (p. 393)

As this quotation makes clear, proponents of embedded cognition are keen to emphasize the way in which (human) cognitive states and processes depend on aspects of the extra-organismic environment, even though they reject the claim that such resources should be seen as part of the physical fabric that *realizes* such states and processes.

This is not the place to revisit arguments regarding the relative merits of extended versus embedded conceptions of cognition (see Clark 2008, 2007a, 2010). What is important, in the present context, is the extent to which the notion of embedded cognition helps to improve our understanding of the space of possibilities concerning the design and implementation of Internet-situated forms of machine intelligence. In fact, as is noted by Clark (2008, p. 139), we should not see the choice between embedded and extended cognition as a “zero-sum game.” Instead, we should see the embedded and extended perspectives as conceptual lenses that are “apt to draw attention to certain features, regularities, and contributions while making it harder to spot others or to give them their problem-solving due” (Clark 2008, p. 139). With this in mind, in what follows, I will attempt to highlight a number of areas where I believe the embedded approach is to be preferred over the extended alternative, specifically when it comes to issues of Internet-situated machine intelligence.

One situation in which the human social environment plays an important role in shaping the course of machine-based processing is to be found in the context of collaborative filtering systems. Consider, for example, the approaches adopted by companies such as Amazon and Netflix with regard to their product recommendation systems. In both cases, there is a significant problem to be solved, namely, the identification of those resources that might appeal to users based on their history of purchasing behaviour. One approach to this problem is to engage in a detailed analysis of each resource, attempting to judge the relative similarity of each resource with respect to a number of dimensions of interest. This, it should be clear, is a task that is of sufficient scale and complexity as to challenge the current state-of-the-art in machine intelligence. The alternative approach is, of course, the one embraced by both Amazon (Linden et al. 2003) and Netflix (Amatriain 2012): simply monitor the purchasing behaviour of the relevant user community and rely on the use of collaborative filtering techniques to make future product recommendations.

What we encounter in the case of collaborative filtering, I suggest, is a situation in which particular kinds of machine intelligence lean very heavily on the human social environment. However, in this particular case, it does not seem appropriate to regard the human social environment as an intrinsic part of the physical mechanisms that realize the target task (i.e., the derivation of product recommendations). Instead, it seems far more appropriate to see the human social environment as (e.g.) generating representations that productively re-shape and re-configure the nature of the computational problem that needs to be tackled by the technological system.

Examples of this sort of (socially-)embedded machine intelligence are, in fact, relatively commonplace on the Internet. Google’s PageRank algorithm (Brin and Page 1998), for example, which sits at the heart of its market-leading search capability, is grounded in the linking behaviour of the Web user community: as links accrue to particular resources, they provide an indication of the worth, interest or value of those resources with respect to subsequent information retrieval activities. In the absence of this sort of structural configuration and enrichment of the online environment, the PageRank algorithm would not be able to support the ranking and sorting capabilities that are exhibited by the search engine. Similarly, when it comes to judgments about the reliability of particular bodies of online information, the endorsement activities of human users (in the form of, for example, explicit user ratings) provides an important source of information that can be factored into credibility evaluations (Taraborelli 2008).

The moral to emerge from these sorts of cases is that the human social environment can often play an important (perhaps indispensable) role in enabling some form of (synthetic) intelligent processing capability to emerge. Unlike the case of HEMC, the human agents in these cases do not seem to be actively involved in the realization of task-relevant information processing routines. Instead, they seem to engage in a productive re-configuration or enrichment of the environment in which machine-based processing occurs. Such a view is reminiscent of the claims made by David Kirsh in respect of human problem-solving (Kirsh 1995, 2009). Kirsh thus suggests that human agents engage in the active structuring of their environments as a means of simplifying or transforming the nature of the problem that confronts the biological brain. Something similar can perhaps be said to occur in the aforementioned cases of product recommendation, resource ranking and reliability evaluation. In such cases, the human social environment can be seen to play a productive role in simplifying and transforming the kind of problem that needs to be solved by some form of synthetic intelligent system. The nature of this dependency, it should be clear, is significant. Absent the human contributions and the performance of the machine-based system is likely to suffer as a result. Indeed, in many cases, the nature of the computational task that confronts the machine-based system is one that can *only* be achieved with the sort of support (deliberate or otherwise) that is provided by the (Internet-using) human community.^7^

## Scaffolded Cognition

Scaffolded cognition, as it name suggests, is a form of cognition in which an array of extra-organismic elements are seen to play an important role in the development of certain kinds of cognitive capability. The term has its roots in the developmental psychology literature, most notably the work of the Soviet psychologist, Lev Vygotsky (1986). Vygotsky’s contribution was to recognize the role played by extra-organismic resources, especially the human social environment, in shaping the developmental profile of a young human infant. The core idea was that the provision of support at crucial points in the developmental trajectory of a young child would eventually enable the child to achieve success in the absence of such support. The support provided by an adult caregiver while a young infant takes her first few faltering steps is one example of this sort of scaffolded development.

There is, of course, a certain sense in which scaffolded cognition appears similar to both extended and embedded cognition. As with the notions of extended and embedded cognition, scaffolded cognition is a form of cognition that is seen to rely on a surrounding nexus of extra-organismic elements. What distinguishes scaffolded cognition from both extended and embedded cognition, however, is the role played by the extra-organismic elements in the *development* of cognitive capabilities. In the case of scaffolded cognition, a supportive framework is erected around a developing structure or capability in order to support its full emergence. Once the capability is fully developed, the scaffolding is no longer required and can be dispensed with. This, I suggest, marks an important point of difference between the notions of scaffolded cognition on the one hand and the notions of extended and embedded cognition on the other. In the case of scaffolded cognition, the environmental resources are helping to alter and inform the development of a set of biologically-based cognitive processing routines—routines that will, at some later stage, emerge as free-standing. This is not something that the proponent of either extended or embedded cognition needs to commit to. In the case of extended cognition, for example, the function of bio-external resources is not to scaffold the development of a biologically-based processing ability (although that may very well emerge as one of the side-effects of some form of cognitively-potent bio-technological union); neither is it to help bring about a future state-of-affairs in which the bio-external resources are no longer required for the runtime realization of some form of cognitive competence. Rather, the purpose of cognitively-potent bio-technological mergers is often to give birth to a new kind of capability, one that is perhaps forever beyond the developmental reach of the biologically-based cognizer.

Analogues to human-based forms of scaffolded cognition can, I suggest, be found in the literature pertaining to Internet-situated machine intelligence. This is not, in fact, as difficult as it might appear, for we have already seen how the Internet provides the basis for ever-more intimate forms of cognitive contact with the human social environment (see Sect. 2), and it is typically elements of the human social environment (i.e., individual human agents) that provide the crucial forms of support in the case of conventional (i.e., human-centered) forms of scaffolded development (recall the role of human caregivers in supporting the infant’s early ambulatory endeavors).^8^ What we are after in the case of machine-based scaffolded cognition, therefore, is a state-of-affairs in which human agents are providing a form of temporary support to the development of some ‘mature’ machine-based cognitive capability. An example of this can be found, I suggest, in the literature on citizen science systems (Lintott and Reed 2013). Consider, for example, a citizen science system known as Galaxy Zoo. Galaxy Zoo is a system in which human participants are asked to identify and classify galaxies based on images from the Sloan Digital Sky Survey (Lintott et al. 2008). The basis for human involvement, in this case, relates to the difficulty of identifying the taxonomically-relevant features of galaxies from a set of 2D images. Recognizing that a particular galaxy is a spiral galaxy, for example, requires an ability to detect an array of morphological features from a complex (and highly variable) set of visual cues. Humans, as it turns out, are quite good at this task, but the task presents something of a challenge for machine-based vision systems. Progress in machine vision tasks often comes about as the result of the application of machine learning techniques to large bodies of appropriately labeled training data. In the case of many machine vision tasks, however, the relevant body of training data is simply not available. The task of galaxy classification is no exception here. But now notice something important: by virtue of the classification efforts of human volunteers, the raw data for the galaxy classification task (i.e., the set of 2D images) is altered. In place of a set of unlabeled images, we now have a growing corpus of images that are reliably associated with taxonomically-relevant labels. The availability of this ‘semantically-enriched’ dataset opens up a range of, previously unavailable, opportunities for machine-based processing. One such opportunity is, of course, to exploit the annotated image set in the context of machine learning techniques that aim to enhance the performance of automated image classifiers. This is precisely the strategy adopted by Dieleman et al. (2015). Dielemen et al. sought to apply a novel class of neural networks, called deep convolutional neural networks, to the galaxy classification task.^9^ Here, the availability of the training dataset, originating from the actions of Galaxy Zoo volunteers, was instrumental in supporting the emergence of a novel form of machine-based intelligent processing capability—one that we can (and should) recognize as a genuine case of machine cognition, and one which (in my view) also represents a *bona fide* case of scaffolded cognitive development.^10^

The importance of human contributions to the development of machine intelligence is explicitly recognized by those who seek to incorporate human subjects into computationally-difficult tasks. With respect to citizen science systems, for example, Lintott and Reed (2013) note that one of the limiting factors in the development of automated processing solutions is the availability of sufficiently well-structured training datasets and that one of the key advantages of citizen science projects is the provision of such datasets. Similarly, when it comes to a class of systems known as Games With A Purpose (GWAP),^11^ von Ahn and Dabbish (2008) are keen to stress the role of human contributions in giving rise to ever-more intelligent forms of machine-based processing:

By leveraging the human time spent playing games online, GWAP game developers are able to capture large sets of training data that express uniquely human perceptual capabilities. This data can contribute to the goal of developing computer programs and automated systems with advanced perceptual or intelligence skills. (von Ahn and Dabbish 2008, p. 67)

In some cases, the machine-based elements can even play an active role in shaping the course of their own cognitive development. They do this by soliciting particular kinds of input from the human user community. A good example of this kind of ‘self-structured machine learning’ or ‘active learning’ (see Settles 2012) is a system described by Barrington et al. (2012). The system in question uses a socially-oriented online game, called Herd It, in which groups of human individuals annotate a musical resource with descriptive tags. These annotations are subsequently used to train a supervised machine learning system that ultimately aims to perform the annotation task independently of the human agents. All this, of course, is broadly consistent with the general profile of machine learning. But what makes Barrington et al.’s system of particular interest is the way in which the machine *directs *the course of its own learning. It does this by actively selecting the musical resources that will be the focus of tagging efforts by the human game players. This is important, because it gives the machine an opportunity to select those forms of feedback that are likely to be of greatest value in terms of informing its subsequent cognitive development. In the words of Barrington et al. (2012), “the machine learning system actively directs the annotation games to collect new data that will most benefit future model iterations” (p. 6411).

The cases of Galaxy Zoo and Herd It are thus compelling examples of how human contributions can play an important, and perhaps indispensable, role in the development of machine-based capabilities. Such cases should, I suggest, be viewed as the machine-based counterparts of the more conventional cases of scaffolded cognition that are discussed in the developmental psychology (Vygotsky 1986) and cognitive science (Sterelny 2010) literatures.

The scaffolding of machine-based capabilities helps to highlight the transformative potential of the Internet with respect to the realization of new kinds of machine cognition. In this respect, the conceptual value of scaffolded cognition is similar to that identified in the case of extended cognition. In both cases, we are able to see how the Internet enables the human social environment to extend the reach of machine-based cognitive capabilities. In the case of extended cognition, the focus, of course, is on the extent to which human agents can be incorporated into the cognitively-relevant processing routines of a machine-based system. In view of the current discussion of scaffolded cognition, however, it should be clear that the transformative impact of the Internet is not exhausted by these sorts of intimate symbiotic cognitive unions. We can also now appreciate the role of online social participation in shaping the developmental trajectory of (what are ultimately) free-standing machine-based capabilities. Once we recognize this possibility, it potentially alters the way that we approach the design and development of online systems. Thus rather than simply designing systems with the aim of exploiting human capabilities for the purposes of performing an otherwise intractable task, we can *also* think about the way in which certain kinds of human inputs may function as a form of cognitive scaffolding, working to enhance machine-based capabilities to the point at which human input is no longer required. This, I suggest, is one of the virtues of viewing the Internet through the conceptual lens of scaffolded cognition: it enables us to review our approach to system design in the light of a new-found appreciation of the sorts of opportunities the Internet provides for the development of future forms of machine intelligence.^12^

## Embodied Cognition

Issues of material embodiment and environmental embedding typically find their greatest expression in an important and influential body of research that goes under the heading of ‘embodied cognition’. Although there are a number of different views as to what is implied by the term ‘embodied cognition’, a common feature of embodied cognition research is the emphasis that is placed on extra-neural bodily factors in shaping the course of cognitive processing (Anderson 2003; Shapiro 2007, 2011). Research into embodied cognition thus typically focuses on the way in which an organism’s bodily structure or physical actions help to constrain (and sometimes constitute) cognition. A somewhat trivial example is provided by the way in which the placement of an organism’s sensory apparatus (e.g., the position of their eyes and ears) helps to structure incoming sensory information in ways that support perceptual processing (Webb 1996). Other forms of embodied cognition research seek to evaluate the role of physical actions (e.g., hand gestures) in supporting various forms of human cognitive competence (Goldin-Meadow 2003; Goldin-Meadow and Beilock 2010; Cook et al. 2008; Andrade 2010).

In the case of biological agents, the body would seem to be a natural focus of attention for those embracing a situated and ecologically-oriented approach to cognition. But do any of the insights gleaned from embodied cognitive science have any relevance to contemporary research on machine intelligence, especially when it comes to the various species of intelligent system that inhabit the ecological niche of the Internet?^13^

One answer to this question comes from recent work on what is referred to as the ‘global brain’ (Heylighen 2013; Kyriazis 2015; Vidal 2015). The global brain, in this case, is a metaphor for a form of planetary-scale superintelligence that is “formed by all people on this planet together with their artifacts and technologies” (Heylighen 2013, p. 905). The idea, in essence, is that global information and communication networks, such as the contemporary Internet, serve as (part of) the realization base for “problem-solving abilities...[that are] orders of magnitudes larger than that of any single individual, organization, or computer system” (Heylighen 2013, p. 905). Crucially (although somewhat confusingly given their appeal to neurological metaphors), global brain theorists accept the idea that intelligence is constituted by more than just the operation of ‘brain-based’ processes. They thus accept the claim (which lies at the heart of the theoretical agenda of embodied cognitive science) that corporeal elements can (and typically will) play an important role in supporting the emergence of intelligent performances (see Heylighen 2012). This much is clear from the way in which proponents of the global brain talk about the contribution of human and technological elements to ‘embodied intelligence’:

The accelerating incorporation of a multitude of hardware sensors and effectors (e.g. satellites, cameras, remote controls) together with an ever more intimate individual-net interface turn the global brain into a truly situated and embodied intelligence...Every human being or piece of machinery hooked up to the web forms part of its body, providing it with additional capabilities for input, output and control. (Heylighen 2012)

Here, then, we encounter one way in which issues of embodied cognition might be able to establish a useful foothold in discussions relating to Internet-situated forms of machine intelligence. The general idea is that, in at least some cases, it may be appropriate to see technological devices (and perhaps even human agents) as literal body parts, i.e., as physical elements that play the same sort of functional role—e.g., the mediation of real-world sensorimotor engagements—that we typically ascribe to conventional (biological) body parts (see Clark 2007b). Inasmuch as such claims are true, we are provided with an important opportunity to consider how issues of embodiment might affect the attempt to engineer online forms of machine intelligence. An important issue for future research is thus to determine whether some of the insights gleaned from research into biological (and robotic) systems can be applied to the realm of online systems and services. This, I suggest, should attempt to go beyond the rather simple case of informational ‘pickup’, where some Internet-accessible resource is used to provide a form of ‘perceptual’ contact with the real-world environment.^14^ In fact, I suggest that the most compelling case for an embodied cognitive science of Internet-situated machine intelligence is likely to come about as a result of the attempt to allow machine-based systems to exert some form of control over their ‘perceptual’ environments. One example might be the case of sensor devices being actively moved and configured in order to structure the flow of incoming sensory information. Another might be the case of human subjects being actively recruited—perhaps via crowdsourcing techniques (Doan et al. 2011)—to engage in tasks that involve the transformation of problematic bodies of target data (e.g., photorealistic images) into something more suitably aligned with the idiosyncratic processing capabilities of a symbol-crunching computational machine.^15^

## Ecological Engineering

Throughout this paper, I have sought to draw on the idea that the Internet forms an important part of the cognitive ecology for artificial cognitive systems. There are a variety of reasons to think that this sort of ecological approach to the Internet is useful, not least because it helps to support the kinds of analyses that were undertaken in respect of extended, embedded, scaffolded and embodied cognition. Now that these analyses have been undertaken, it is time to highlight an unexpected bonus of the ecological perspective. This bonus comes into view once we pay attention to the emphasis that is placed on the notions of ecological engineering (Sterelny 2003) and cognitive niche construction (Clark 2008) in contemporary cognitive science. The core idea, in brief, is that biological agents can sometimes play an active role in creating and configuring their environments in ways that work to structure, support and sustain their cognitive processing routines. In respect of the notion of cognitive niche construction, for example, Clark (2008) suggests that we should see cognitive niche construction as “the process by which animals build physical structures that transform problem spaces in ways that aid (or sometimes impede) thinking and reasoning about some target domain or domains” (p. 62). Such processes are deemed to be important because they provide a powerful means of bootstrapping certain kinds of cognitive capability. In the case of extended cognition, for example, we humans have the opportunity to create the very resources that we subsequently assimilate into our cognitive processing routines. We thus have to the ability to engage in an important form of cognitive ‘self-engineering’, effectively creating the conditions under which we are able to the extend the reach of our cognitive capabilities. “We use intelligence,” writes Clark, “to structure our environment so that we can succeed with less intelligence. Our brains make the world smart so that we can be dumb in peace!” (Clark 1997, p. 180).

Perhaps unsurprisingly, the notion of cognitive niche construction is just as applicable to the realm of the online environment as it is to the realm of the offline (or real-world) environment. This is particularly so once we consider the nature of our interactions and engagements with the contemporary Internet. With the advent of what is sometimes referred to as Web 2.0, we have witnessed a transition in the way human agents interact with the Web. Thus, in addition to merely consuming online content (as was the case with earlier versions of the Web), human Internet users are now in a position to create and configure a significant amount of the content that comprises the informational ecology of the online world. This is perhaps most evident in the case of systems that are referred to as ‘social machines’ (Hendler and Berners-Lee 2010). These are systems that, according to Hendler and Berners-Lee (2010, p. 156), enable the relevant user community to participate in the creation and editing of online content. Wikipedia serves as one example of this class of systems. In this case, the majority of the online content (i.e., the content of Wikipedia articles) is contributed by members of the human user community.^16^ Other prominent examples include social networking systems, such as Facebook, microblogging services, such as Twitter, and social media sites, such as YouTube and Flickr. Inasmuch as these systems can be seen as part of the cognitive ecology of the Internet, then it seems appropriate to see ourselves (both individually and collectively) as engaged in a process of ‘online ecological engineering’ or ‘online cognitive niche construction’, actively creating and configuring the environment that influences the profile of our subsequent cognitive and epistemic endeavours.^17^

But it is not only human cognition that stands to be affected by this particular form of ecological engineering. If the arguments rehearsed in this paper are anywhere near the mark, then it becomes clear that we should see the actions of human agents as not only altering the ecological setting for human intelligence but as also affecting the environment in which various forms of machine intelligence are materially embedded. We thus begin to creep up on an idea that first surfaced in the discussion of scaffolded cognition (see Sect. 5). This is the idea that the Internet affords a relatively unique opportunity for the global human community to create the sorts of conditions under which advanced forms of machine intelligence might emerge. Contrary to any radical sea-change in our approach to machine learning, or some dramatic breakthrough in our understanding of the mechanistic origins of intelligent behavior, perhaps what is really required to advance the state-of-the-art in machine intelligence is merely(!) a means of creating the sort of ecological niche that enables machine-based cognitive systems to thrive and flourish. Our online interactions, if this is correct, might be seen as a means of progressively shaping (and re-shaping) the specific mix of opportunities and affordances that help to bring the next generation of cognitive computing systems into being.

If this all seems somewhat vague, perhaps it will help to consider a concrete example of how human activity, when appropriately supported and enabled by the technological infrastructure of the Internet, can lead to advances in machine intelligence. Take IBM Watson, the poster child of the emerging cognitive computing paradigm (Kelly and Hamm 2013). Watson’s virtuoso performance in answering of array of difficult questions is undoubtedly an important demonstration of the growing sophistication of machine-based processing, especially in the areas of natural language processing, machine learning and domain-specific reasoning. In the course of being awestruck by (at least some of) Watson’s outputs, however, it is easy to overlook the simple fact that many of the inputs to the system—especially the information resources that Watson exploits in supplying its human interlocutors with answers—are ones that are, in general, developed by large numbers of human individuals. Such resources include online encyclopedias, dictionaries, thesauri, taxonomies, ontologies and so on (Ferrucci et al. 2010). None of these resources, it should be clear, were specifically intended to support Watson, or indeed any of the other cognitive computing systems that are the focus of current research interest. Rather, what has happened is that as a result of the Internet’s ability to support and solicit online engagement we now have access to an array of resources that can be used to fuel the development of relatively novel forms of machine intelligence. If such systems—I am thinking primarily of Watson here—had appeared in the absence of the Internet, we would, I suspect, have been astonished at their capabilities. And yet Watson is, at root, exactly the same kind of symbol manipulating device that has long been the focus of philosophical theorizing and the primary instrument of cognitive scientific practice. Arguably, what has changed in recent times is not so much the underlying technology, as the nature of the environment in which such technologies are situated. The Internet has thus enabled us to create the sort of environment in which a conventional symbol crunching computational economy is able to exhibit forms of intelligence that might have seemed utterly out of reach just a few short years ago.

The key take home message of this section is that it sometimes makes sense to see intelligence as (in part) a product of the environment in which an agent is materially embedded. Inasmuch as this is true, and inasmuch as it applies to the case of machine intelligence, then it clearly makes sense to see issues of environmental structuring, enrichment and configuration as of crucial importance to the enhancement of machine-based cognitive capabilities. Given that the human-engineered environment is deemed to be of crucial relevance to our own human cognitive capabilities (Clark 2003, 2008), why not assume that the key to advanced forms of machine intelligence is likewise to be found in an environment that is home to a growing number of our intelligent machines?

## Conclusion

In the present paper, I suggested that we should view the Internet as a form of cognitive ecology. The primary value of this perspective is that it helps us gain a better understanding of how the Internet might shape and influence the profile of machine-based cognitive capabilities, both now and in the future. The idea that the Internet forms part of the cognitive ecology for machine intelligence also brings a range of other benefits. For example, it enables us to establish a useful point of contact with work that emphasizes an ecological approach to *human* cognition (Hutchins 2010; Malafouris 2013; Tribble and Sutton 2011; Bateson 1972). It should also be clear that an ecological approach enables us to take a number of different conceptual stances when it comes to an analysis of the cognitive significance of the Internet. We can thus view the Internet from the perspective of those approaches that are typically associated with the situated cognition movement, e.g., extended, embedded, scaffolded and embodied cognition (see Robbins and Aydede 2009b). When we apply this situated perspective to the case of Internet-based forms of machine intelligence, a number of interesting and important issues come to light. These include, but are not necessarily limited to, the following:

- *Philosophical issues* As we saw in the discussion of HEMC (see Sect. 2), a situated approach to machine intelligence can help to reshape and reorient the trajectory of existing philosophical debates. When it comes to extended cognition, for example, much of the philosophical discourse is based around the idea of human-centered—or organism-centered (see Clark 2008)—cognition. By applying the notion of extended cognition to the realm of machine intelligence, we help to broaden the philosophical debate and (hopefully) instigate new forms of philosophical progress.
- *Engineering issues* A situated approach is also useful in helping to inform the design and development of online systems. We saw, for example, that manually annotated resources can sometimes be used to support the development of fully automated capabilities (see Sect. 4). Once we appreciate this possibility, it potentially alters the way that we approach the design and development of future systems.
- *Cognitive science issues* In the context of the discussion on collaborative filtering (see Sect. 3), we encountered the idea that the human social environment could be exploited as a form of computationally-significant resource. This is an idea that seems to be gaining increasing traction within the computer science community (Bernstein et al. 2012; Robertson and Giunchiglia 2013). In the extreme case, we can perhaps see the Internet as transforming the human social environment into something of a ‘social computer’—a large scale socio-computational system that is able to shape, support and sustain the next generation of AI systems.
- *Ethical issues* Once we recognize the role of human-Internet interactions in the development and operation of machine-based cognitive processing routines, a number of important ethical issues start to come to light. Such issues are particularly apparent if we accept that human agents can form part of the physical machinery that realizes machine-based cognitive processes (see Sect. 2). In this case, it is natural to wonder whether the support for machine cognition is predicated on a form of technological control reminiscent of that exerted by the factory-based machines of the Industrial Era (see Black 2014). There is also an important issue hereabouts concerning the extent to which it is appropriate for human agents to be incorporated into processes that they themselves might regard as ethically unacceptable.
- *Economic issues* On a somewhat related note, many of our contributions to the online cognitive ecology are seldom subject to financial remuneration. This raises an important issue concerning the extent to which the (financial) beneficiaries of machine-based cognitive systems (e.g., major technology vendors) should be allowed to capitalize on what is, in effect, a form of unpaid ‘digital labour’.

As should be clear from this list, the kinds of issues raised by the present paper reach well-beyond the disciplinary borders of cognitive science. We can thus begin to see how a situated approach to machine intelligence, in conjunction with a cognitive ecological approach to the Internet, helps to bring an important array of issues into the scientific, social, philosophical and engineering spotlight.

From a purely cognitive science perspective, the primary value of viewing the Internet as a form of cognitive ecology is that it helps us to appreciate the transformative potential of the Internet vis-à-vis future forms of (human and) machine intelligence. Given the way in which an ecological perspective encourages us to see cognition as heavily dependent on, if not partly constituted by, aspects of the wider environment, we can view the Internet as marking a potentially significant waypoint in the ‘evolution’ of machine-based capabilities. We have seen, for example, how the Internet provides an unprecedented opportunity to tap into human capabilities in a manner and on a scale that would have been unthinkable just a couple of decades ago (see Sect. 2). We have also encountered the idea that the Internet provides access to a variety of kinds of training data that are progressively altering our traditional approaches to machine learning (see Sect. 4). Finally, we have seen how the Internet affords access to bodies of information that can be used to develop intelligent systems whose capabilities are, at least in some respects, superior to those of their human counterparts (recall the discussion of IBM Watson in Sect. 6).

The advent of the Internet thus marks a potentially important milestone in our attempts to engineer future forms of machine intelligence. In helping us to press maximal cognitive benefit from this new environment, we should, I suggest, seek to establish intellectual contact with work in the philosophy of mind that aims to illuminate the ways in which human cognition is influenced by an array of extra-neural and extra-organismic resources. Such points of contact may be invaluable in terms of helping us to appreciate the many ways in which the Internet helps to shape the intelligence of both ourselves and the machines we build.

## Footnotes

1 The term ‘Internet’ is used to refer to the global system of information and communication networks that rely on Internet communication protocols to achieve end-to-end data transfer. For the purposes of the present paper, the term ‘Internet’ is used as a convenient way of referring to all the various applications that are built on top of the Internet protocol suite. These include the World Wide Web (or Web), which relies on the use of a specific protocol, namely, HyperText Transfer Protocol (HTTP). The term is also used to refer to a number of other applications, including email, peer-to-peer networking, the Internet of Things and so on.

2 I will assume that at least some forms of machine-based processing activity can be viewed as *bona fide* forms of cognitive processing. The current interest in cognitive computing systems (Hurwitz et al. 2015; Kelly and Hamm 2013; Modha et al. 2011) is testament to the fact that at least *some* forms of machine-based processing can be (and typically are) viewed in cognitive terms.

3 This is because a situated approach to cognition enables us to focus on the role of the extra-organismic and extra-neural environment (i.e., the cognitive ecology) in shaping the cognitive capabilities of the biological or (in this case) the technological agent.

4 It should be noted that this review is not exhaustive, in the sense that it does not attempt to consider a number of other forms of cognition that could be subsumed under the banner of situated cognition. There is, in particular, no attempt to cover the considerable body of work relating to enactive cognition (Stewart et al. 2010; Engel et al. 2016; Froese et al. 2011), grounded cognition (Barsalou 2008, 2010; Pezzulo et al. 2013; Pecher and Zwaan 2005) and collective/distributed cognition (Hutchins 1995). It should be clear that the Internet is relevant to at least some of these additional forms of situated cognition. When it comes to collective/distributed cognition, for example, the Internet provides the basis for cognitively-potent forms of ‘social’ interaction and engagement between collections of purely synthetic agents. This can be seen as a potential boon for research that goes under the heading of distributed AI (see Clearwater et al. 1992).

5 For example, we can imagine human biological brains beng incorporated into complex computational tasks by virtue of the use of advanced brain activity recording devices or imaging techniques. The use of such techniques in the context of rapid serial visual presentation tasks provides one example of such forms of bio-technologically hybrid processing (see Huang et al. 2011).

6 There is, of course, an important issue hereabouts concerning the extent to which humans agents are actually willing to participate in machine-based processing. The mere fact that almost everyone on the planet can connect to the Internet does not mean that anyone will actually be ‘available’ for incorporation into a particular information processing routine. Issues of incentivization and motivation are clearly important here, as is the extent to which the temporal profile of human participation can be aligned with the temporal demands of the target task. With respect to issues to timing, it is important to note that the real-time recruitment and engagement of human participants into some form of information processing activity is a topic that is the focus of current research in the human computation and crowdsourcing communities. Research in this area typically goes under the heading of real-time or continuous crowdsourcing (Lasecki and Bigham 2013).

7 Note that the majority of human interactions with the Internet are not ones that are intended to support machine cognition. Instead, in many cases, the support for machine cognition comes about as an indirect result of our everyday interactions and engagements with the online environment.

8 This should not, of course, blind us to the possibility that machine intelligence might be scaffolded in ways that do *not* involve human agents. There might, for example, be cases where the development of machine-based cognitive capabilities is scaffolded by other kinds of technological system.

9 Convolutional neural networks are a form of deep learning architecture that is commonly used for image processing tasks (Bengio 2009). Although not all deep learning architectures require appropriately labeled training data, the Internet has clearly played an important role in securing access to the sort of datasets that are typically used to train deep learning systems.

10 It should be clear, for example, that the human contributions are playing an important (and perhaps indispensable) role in supporting the emergence of what is ultimately a free-standing capability. There is, in addition, no reason why we should fail to recognize this as a genuine case of machine cognition. We can thus ask ourselves whether the machine-based capability is one that we would be content to accept as cognitive were it to be manifest by a human agent. The result, in this case, is surely positive. After all, the task that is being performed by the machine vision system—i.e., morphological galaxy classification—is more-or-less exactly the same task as that being performed by the human agents who generated the training data. If we accept that classification outcomes in the human case are the result of a *cognitive* ability, why should we regard the results of machine-based processing any differently?

11 GWAPs are computer games that are designed to make use of game player actions (von Ahn 2006; Savage 2012; Cooper et al. 2010b). Often such games are used to support the process of scientific discovery (Good and Su 2011; Khatib et al. 2011b). We thus encounter examples of GWAPs in the domains of neuroscience (Marx 2013), proteomics (Cooper et al. 2010a), astronomy (Lintott et al. 2008), and oncology (Coburn 2014).

12 One example where we see evidence of this sort of influence is in the design of citizen science systems. In the case of PenguinWatch (see https://www.penguinwatch.org/), for example, human volunteers are required to identify the location of penguins in a set of images using a distinctive annotation. Such annotations provide a form of additional structure that can be used to bolster the performance of machine learning algorithms (Grant Miller, personal communication, February 24, 2016).

13 Claims about the importance of an embodied perspective in the case of other forms of intelligent system should be largely uncontroversial. This is particularly so when it comes to the study of robotic systems. Consider, for example, some of the early work in robotics where behavioral success was seen to result from the exploitation of simple body-related sensorimotor contingencies. Clark’s (1999) description of the can-collecting robot, Herbert, for example, highlights the role of bodily motion (in this case, the rotation of the robot’s ‘torso’) in securing an appropriate physical alignment between the robot and a target object—one that helps to secure the success of subsequent reaching movements.

14 Some examples derive from research into participatory sensing (Carrera et al. 2013; Resch 2013) and collective sensing (Dugas et al. 2013; Ginsberg et al. 2009; Resch 2013). In the case of collective sensing, for example, the inputs provided by human agents can sometimes be used to support the detection of phenomena that are otherwise very difficult to discern. A notable example, in this case, concerns the use of query-based syndromic surveillance techniques to monitor trends in disease pandemics. By monitoring the prevalence of semantically-relevant Google search terms, it has thus proved possible to detect and monitor the progress of influenza outbreaks (Dugas et al. 2013; Ginsberg et al. 2009). Similarly, the analysis of online search behaviour has yielded insights into the seasonal nature of mental health concerns (Ayers et al. 2013), as well as information about the side-effects of particular medicines (White et al. 2013).

15 Such human-mediated transformations of the target data could assume a variety of forms. One example is provided by systems that support the (human-assisted) semantic annotation of complex resources, such as medical X-ray images (see Rubin et al. 2008). These sorts of interventions are typically aimed at improving machine-based access to important bodies of task-relevant information, many of which are ‘hidden’ or ‘deeply embedded’ in the focal resource. Human annotations, in this case, help to resolve what is sometimes referred to as the ‘semantic gap’ when it comes to the analysis of online content (see Siorpaes and Simperl 2010).

16 Note that even if the actual number of contributors (e.g., Wikipedia editors) is relatively small compared to the total size of the relevant user community (e.g., the total number of individuals that access Wikipedia), it is still true to say that much of the online content is being generated by the human ‘users’ of the system.

17 Part of this process, it should be clear, involves the design and development of the systems (e.g., Facebook) that we interact with whenever we go online. In this case, it seems that we play (at best) only a minor role in the ‘engineering’ process, indirectly influencing design and development decisions via our effect on market forces. There is, however, another sense in which we are poised to play a more active and direct role in the process of online ecological engineering. This becomes clear when we reflect on the way in which every form of human-Internet interaction is able to alter the informational content and computational dynamics of the online environment. Thus every time we perform a Google Search, we provide a body of data that can be used for a variety of purposes, for example, delivering personalized search results (Simpson 2012) or implementing query-based syndromic surveillance systems (Dugas et al. 2013). The same applies to purchasing decisions on Amazon (recall the discussion of collaborative filtering in Sect. 3). Thus every time we make a purchase using Amazon, we provide a form of data that is poised to influence the kind of informational contact that both ourselves and others have with the online environment. These examples help us see that our role as ecological engineers is not limited to the rather straightforward case of (e.g.) editing a Wikipedia article or uploading a YouTube video. Instead, we can (and should) think of even somewhat subtle forms of interaction and engagement with the online environment (e.g., watching a YouTube video) as constituting a cognitively- and epistemically-potent form of online ecological engineering.

## References

[CR3] Amatriain X (2012). Mining large streams of user data for personalized recommendations. ACM SIGKDD Explorations Newsletter.

[CR4] Anderson M (2003). Embodied cognition: A field guide. Artificial Intelligence.

[CR5] Andrade J (2010). What does doodling do?. Applied Cognitive Psychology.

[CR6] Ayers JW, Althouse BM, Allem JP, Rosenquist JN, Ford DE (2013). Seasonality in seeking mental health information on Google. American Journal of Preventive Medicine.

[CR7] Barrett L (2011). Beyond the brain: How body and environment shape animal and human minds.

[CR8] Barrington L, Turnbull D, Lanckriet G (2012). Game-powered machine learning. Proceedings of the National Academy of Sciences.

[CR9] Barsalou L (2008). Grounded cognition. Annual Review of Psychology.

[CR10] Barsalou LW (2010). Grounded cognition: Past, present, and future. Topics in Cognitive Science.

[CR11] Bateson G (1972). Steps to an ecology of mind.

[CR12] Bengio Y (2009). Learning deep architectures for AI. Foundations and Trends in Machine Learning.

[CR13] Berners-Lee T, Hendler J, Lassila O (2001). The Semantic web. Scientific American.

[CR14] Bernstein A, Klein M, Malone TW (2012). Programming the global brain. Communications of the ACM.

[CR15] Black D (2014). Where bodies end and artefacts begin: Tools, machines and interfaces. Body & Society.

[CR16] Branson S, Horn G, Wah C, Perona P, Belongie S (2014). The ignorant led by the blind: A hybrid human–machine vision system for fine-grained categorization. International Journal of Computer Vision.

[CR17] Brin, S., & Page, L. (1998). The anatomy of a large-scale hypertextual search engine. In *7th Annual Conference of the World Wide Web*, Brisbane, Australia.

[CR18] Carrera, F., Guerin, S., & Thorp, J. (2013). By the people, for the people: The crowdsourcing of “STREETBUMP”: An automatic pothole mapping app. *International Archives of the Photogrammetry, Remote Sensing and Spatial Information Sciences*, XL-4/W1, 19–23.

[CR19] Clark A (1997). Being there: Putting brain, body and world together again.

[CR20] Clark A (1999). Where brain, body, and world collide. Cognitive Systems Research.

[CR21] Clark A (2003). Natural-born cyborgs: Minds, technologies and the future of human intelligence.

[CR22] Clark A (2007). Curing cognitive hiccups: A defense of the extended mind. Journal of Philosophy.

[CR23] Clark A (2007). Re-inventing ourselves: The plasticity of embodiment, sensing, and mind. Journal of Medicine and Philosophy.

[CR24] Clark A (2008). Supersizing the mind: Embodiment, action, and cognitive extension.

[CR25] Clark A (2010). Much ado about cognition. Mind.

[CR26] Clark A, Chalmers D (1998). The extended mind. Analysis.

[CR27] Clearwater SH, Hogg T, Huberman BA, Huberman BA (1992). Cooperative problem solving. Computation: The micro and the macro view.

[CR28] Clowes R (2015). Thinking in the cloud: The cognitive incorporation of cloud-based technology. Philosophy & Technology.

[CR29] Coburn C (2014). Play to cure: Genes in space. Lancet Oncology.

[CR30] Cook SW, Mitchell Z, Goldin-Meadow S (2008). Gesturing makes learning last. Cognition.

[CR31] Cooke NJ, Gorman JC, Rowe LJ, Salas E, Goodwin GF, Burke CS (2004). An ecological perspective on team cognition. Team effectiveness in complex organizations: Cross-disciplinary perspectives and approaches.

[CR32] Cooper S, Khatib F, Treuille A, Barbero J, Lee J, Beenen M, Leaver-Fay A, Baker D, Popović Z, Players Foldit (2010). Predicting protein structures with a multiplayer online game. Nature.

[CR33] Cooper, S., Treuille, A., Barbero, J., Leaver-Fay, A., Tuite, K., Khatib, F., et al. (2010b). The challenge of designing scientific discovery games. In *5th International Conference on the Foundations of Digital Games*, Monterey, California, USA.

[CR34] Dieleman S, Willett KW, Dambre J (2015). Rotation-invariant convolutional neural networks for galaxy morphology prediction. Monthly Notices of the Royal Astronomical Society.

[CR35] Doan A, Ramakrishnan R, Halevy AY (2011). Crowdsourcing systems on the World Wide Web. Communications of the ACM.

[CR36] Dugas AF, Jalalpour M, Gel Y, Levin S, Torcaso F, Igusa T (2013). Influenza forecasting with Google Flu Trends. PLoS One.

[CR37] Engel AK, Friston KJ, Kragic D (2016). The pragmatic turn: Toward action-oriented views in cognitive science.

[CR38] Ferrucci D, Brown E, Chu-Carroll J, Fan J, Gondek D, Kalyanpur AA, Lally A, Murdock JW, Nyberg E, Prager J (2010). Building Watson: An overview of the DeepQA project. AI Magazine.

[CR39] Fogarty, J., Tan, D., Kapoor, A., & Winder, S. (2008). CueFlik: Interactive concept learning in image search. In *SIGCHI Conference on Human Factors in Computing Systems*, Florence, Italy.

[CR40] Froese T, Paolo D, Ezequiel A (2011). The enactive approach: Theoretical sketches from cell to society. Pragmatics & Cognition.

[CR41] Ginsberg J, Mohebbi MH, Patel RS, Brammer L, Smolinski MS, Brilliant L (2009). Detecting influenza epidemics using search engine query data. Nature.

[CR42] Goldin-Meadow S (2003). Hearing gesture: How our hands help us think.

[CR43] Goldin-Meadow S, Beilock SL (2010). Action’s influence on thought: The case of gesture. Perspectives on Psychological Science.

[CR44] Good BM, Su AI (2011). Games with a scientific purpose. Genome Biology.

[CR45] Halpin H (2013) Does the Web extend the mind? In *5th Annual ACM Web Science Conference*, Paris, France.

[CR46] Halpin, H., Clark, A., & Wheeler, M. (2010). Towards a philosophy of the Web: Representation, enaction, collective intelligence. In *Web Science Conference*, Raleigh, NC, USA.

[CR47] Hendler J, Berners-Lee T (2010). From the Semantic Web to social machines: A research challenge for AI on the World Wide Web. Artificial Intelligence.

[CR48] Heylighen F (2012). A brain in a vat cannot break out: Why the singularity must be extended, embedded and embodied. Journal of Consciousness Studies.

[CR49] Heylighen F, Michelucci P (2013). From human computation to the global brain: The self-organization of distributed intelligence. Handbook of human computation.

[CR50] Hirose N (2002). An ecological approach to embodiment and cognition. Cognitive Systems Research.

[CR51] Horvitz EJ (2007). Reflections on challenges and promises of mixed-initiative interaction. AI Magazine.

[CR52] Huang Y, Erdogmus D, Pavel M, Mathan S, Hild KE (2011). A framework for rapid visual image search using single-trial brain evoked responses. Neurocomputing.

[CR53] Hurwitz JS, Kaufman M, Bowles A (2015). Cognitive computing and big data analytics.

[CR54] Hutchins E (1995). Cognition in the wild.

[CR55] Hutchins E, Smelser N, Baltes P (2001). Distributed cognition. International encyclopedia of the social and behavioral sciences.

[CR56] Hutchins E (2010). Topics in cognitive science. Cognitive Science.

[CR57] Jacucci G, Gamberini L, Freeman J, Spagnolli A (2014). Symbiotic interaction.

[CR58] Kapoor, A., Tan, D., Shenoy, P., & Horvitz, E. (2008). Complementary computing for visual tasks: Meshing computer vision with human visual processing. In *8th IEEE International Conference on Automatic Face and Gesture Recognition*, Amsterdam, The Netherlands.

[CR59] Kelly JE, Hamm S (2013). Smart machines: IBM’s Watson and the era of cognitive computing.

[CR60] Kendon V, Sebald A, Stepney S (2015). Heterotic computing: Past, present and future. Philosophical Transactions of the Royal Society of London A: Mathematical, Physical and Engineering Sciences.

[CR61] Khatib F, Cooper S, Tyka MD, Xu K, Makedon I, Popović Z, Baker D, Foldit Players (2011). Algorithm discovery by protein folding game players. Proceedings of the National Academy of Sciences.

[CR62] Khatib F, DiMaio F, Cooper S, Kazmierczyk M, Gilski M, Krzywda S, Zabranska H, Pichova I, Thompson J, Popović Z, Jaskolski M, Baker D (2011). Crystal structure of a monomeric retroviral protease solved by protein folding game players. Nature Structural & Molecular Biology.

[CR63] Kirsh D (1995). The intelligent use of space. Artificial Intelligence.

[CR64] Kirsh D, Robbins P, Aydede M (2009). Problem solving and situated cognition. The Cambridge handbook of situated cognition.

[CR65] Kraut R, Maher ML, Olson J, Malone TW, Pirolli P, Thomas JC (2010). Scientific foundations: A case for technology-mediated social-participation theory. Computer.

[CR66] Kyriazis M (2015). Systems neuroscience in focus: From the human brain to the global brain?. Frontiers in Systems Neuroscience.

[CR67] Lasecki WS, Bigham JP, Michelucci P (2013). Interactive crowds: Real-time crowdsourcing and crowd agents. Handbook of human computation.

[CR68] Law E, von Ahn L (2011). Human computation. Synthesis Lectures on Artificial Intelligence and Machine Learning.

[CR69] Licklider JCR (1960). Man-computer symbiosis. IRE Transactions on Human Factors in Electronics.

[CR70] Linden G, Smith B, York J (2003). Amazon.com recommendations: Item-to-item collaborative filtering. IEEE Internet Computing.

[CR71] Lintott CJ, Reed J, Michelucci P (2013). Human computation in citizen science. Handbook of human computation.

[CR72] Lintott CJ, Schawinski K, Slosar A, Land K, Bamford S, Thomas D, Raddick MJ, Nichol RC, Szalay A, Andreescu D, Murray P, van den Berg J (2008). Galaxy Zoo: Morphologies derived from visual inspection of galaxies from the Sloan Digital Sky Survey. Monthly Notices of the Royal Astronomical Society.

[CR73] Malafouris L (2013). How things shape the mind: A theory of material engagement.

[CR74] Marx V (2013). Neuroscience waves to the crowd. Nature Methods.

[CR75] Menary R (2010). The extended mind.

[CR76] Michelucci P (2013). Handbook of human computation.

[CR77] Michelucci P, Bainbridge W, Roco M (2016). Human computation and convergence. Handbook of science and technology convergence.

[CR78] Michelucci P, Dickinson JL (2016). The power of crowds. Science.

[CR79] Modha DS, Ananthanarayanan R, Esser SK, Ndirango A, Sherbondy AJ, Singh R (2011). Cognitive computing. Communications of the ACM.

[CR80] Najafabadi MM, Villanustre F, Khoshgoftaar TM, Seliya N, Wald R, Muharemagic E (2015). Deep learning applications and challenges in big data analytics. Journal of Big Data.

[CR81] Neisser U (1997). The ecological study of memory. Philosophical Transactions of the Royal Society B: Biological Sciences.

[CR82] O’Leary DE (2013). Artificial intelligence and big data. IEEE Intelligent Systems.

[CR83] Pecher D, Zwaan R (2005). Grounding cognition: The role of perception and action in memory, language, and thinking.

[CR84] Pezzulo G, Barsalou LW, Cangelosi A, Fischer MH, McRae K, Spivey MJ (2013). Computational grounded cognition: A new alliance between grounded cognition and computational modeling. Frontiers in Psychology.

[CR85] Resch B, Krisp JM (2013). People as sensors and collective sensing-contextual observations complementing geo-sensor network measurements. Progress in Location-Based Services.

[CR86] Robbins P, Aydede M (2009). The Cambridge handbook of situated cognition.

[CR87] Robbins P, Aydede M, Robbins P, Aydede M (2009). A short primer on situated cognition. The Cambridge handbook of situated cognition.

[CR88] Robertson D, Giunchiglia F (2013). Programming the social computer. Philosophical Transactions of the Royal Society A: Mathematical, Physical and Engineering Sciences.

[CR89] Rubin, D. L., Rodriguez, C., Shah. P., & Beaulieu, C. F. (2008). iPad: Semantic annotation and markup of radiological images. In *AMIA 2008 Annual Symposium*, Washington DC, USA. PMC2655990 18999144

[CR90] Rupert R (2004). Challenges to the hypothesis of extended cognition. Journal of Philosophy.

[CR91] Rupert R (2009). Cognitive Systems and the Extended Mind.

[CR92] Savage N (2012). Gaining wisdom from crowds. Communications of the ACM.

[CR93] Settles B (2012). Active learning. Synthesis Lectures on Artificial Intelligence and Machine Learning.

[CR94] Shapiro L (2007). The embodied cognition research programme. Philosophy Compass.

[CR95] Shapiro LA (2011). Embodied cognition.

[CR96] Shapiro LA (2014). The Routledge handbook of embodied cognition.

[CR97] Simpson TW (2012). Evaluating Google as an epistemic tool. Metaphilosophy.

[CR98] Siorpaes K, Simperl E (2010). Human intelligence in the process of semantic content creation. World Wide Web.

[CR99] Smart PR (2012). The Web-extended mind. Metaphilosophy.

[CR100] Smart P. R. (2013). Understanding the cognitive impact of emerging Web technologies: A research focus area for embodied, extended and distributed approaches to cognition. In *1st International Web for Wellbeing and Human Performance Workshop*, Paris, France.

[CR101] Smart PR, Shapiro LA (2014). Embodiment, cognition and the World Wide Web. The Routledge handbook of embodied cognition.

[CR102] Smart, P. R. (forthcoming). Emerging digital technologies: Implications for extended conceptions of cognition and knowledge. In A. J. Carter, A. Clark, J. Kallestrup, O. S. Palermos, & D. Pritchard (Eds.), *Extended epistemology*. Oxford: Oxford University Press.

[CR103] Smart PR, Shadbolt NR, Khosrow-Pour M (2014). Social machines. Encyclopedia of information science and technology.

[CR104] Smart, P. R., Heersmink, R., Clowes, R. W. (forthcoming). The cognitive ecology of the Internet. In S. J. Cowley, F. Vallée-Tourangeau (Eds.), *Cognition beyond the brain* (2nd ed.). London: Springer.

[CR105] Staley DJ (2014). Brain, mind and internet: A deep history and future.

[CR106] Sterelny K (2003). Thought in a hostile world: The evolution of human cognition.

[CR107] Sterelny K (2010). Minds: Extended or scaffolded?. Phenomenology and the Cognitive Sciences.

[CR108] Stewart JR, Gapenne O, Di Paolo EA (2010). Enaction: Toward a new paradigm for cognitive science.

[CR109] Sutton J, Menary R (2010). Exograms and interdisciplinarity: History, the extended mind, and the civilizing process. The extended mind.

[CR110] Taraborelli D, Waelbers K, Briggle A, Brey P (2008). How the Web is changing the way we trust. Current issues in computing and philosophy.

[CR111] Theiner G, Shapiro LA (2014). Varieties of group cognition. The Routledge handbook of embodied cognition.

[CR112] Theiner G, Allen C, Goldstone RL (2010). Recognizing group cognition. Cognitive Systems Research.

[CR113] Tribble E, Sutton J (2011). Cognitive ecology as a framework for Shakespearean studies. Shakespeare Studies.

[CR114] Vidal C, Goertzel B, Goertzel T (2015). Distributing cognition: From local brains to global brain. The end of the beginning: Life, society and economy on the brink of the singularity.

[CR1] von Ahn L (2006). Games with a purpose. Computer.

[CR2] von Ahn L, Dabbish L (2008). Designing games with a purpose. Communications of the ACM.

[CR115] Vygotsky LS (1986). Thought and language.

[CR116] Webb B (1996). A cricket robot. Scientific American.

[CR117] White RW, Tatonetti NP, Shah NH, Altman RB, Horvitz E (2013). Web-scale pharmacovigilance: Listening to signals from the crowd. Journal of the American Medical Informatics Association.

[CR118] Wilson RA, Clark A, Robbins P, Aydede M (2009). Situated cognition: Letting nature take its course. The Cambridge handbook of situated cognition.

